# Circulating extracellular vesicles are associated with the clinical outcomes of sepsis

**DOI:** 10.3389/fimmu.2023.1150564

**Published:** 2023-04-25

**Authors:** Pengfei Li, Yan Wu, Andrew J. Goodwin, Bethany Wolf, Perry V. Halushka, Hongjun Wang, Basilia Zingarelli, Hongkuan Fan

**Affiliations:** ^1^ Department of Pathology and Laboratory Medicine, Medical University of South Carolina, Charleston, SC, United States; ^2^ Division of Pulmonary, Critical Care, Allergy, and Sleep Medicine, Medical University of South Carolina, Charleston, SC, United States; ^3^ Department of Public Health Sciences, Medical University of South Carolina, Charleston, SC, United States; ^4^ Department of Medicine, Medical University of South Carolina, Charleston, SC, United States; ^5^ Department of Pharmacology, Medical University of South Carolina, Charleston, SC, United States; ^6^ Departments of Surgery, Medical University of South Carolina, Charleston, SC, United States; ^7^ Division of Critical Care Medicine, Cincinnati Children’s Hospital Medical Center, Cincinnati, OH, United States

**Keywords:** sepsis, extracellular vesicles, caspase-1, miR-126, vascular injury, ARDS (acute respiratory distress syndrome)

## Abstract

**Introduction:**

Sepsis is associated with endothelial cell (EC) dysfunction, increased vascular permeability and organ injury, which may lead to mortality, acute respiratory distress syndrome (ARDS) and acute renal failure (ARF). There are no reliable biomarkers to predict these sepsis complications at present. Recent evidence suggests that circulating extracellular vesicles (EVs) and their content caspase-1 and miR-126 may play a critical role in modulating vascular injury in sepsis; however, the association between circulating EVs and sepsis outcomes remains largely unknown.

**Methods:**

We obtained plasma samples from septic patients (n=96) within 24 hours of hospital admission and from healthy controls (n=45). Total, monocyte- or EC-derived EVs were isolated from the plasma samples. Transendothelial electrical resistance (TEER) was used as an indicator of EC dysfunction. Caspase-1 activity in EVs was detected and their association with sepsis outcomes including mortality, ARDS and ARF was analyzed. In another set of experiments, total EVs were isolated from plasma samples of 12 septic patients and 12 non-septic critical illness controls on days 1, and 3 after hospital admission. RNAs were isolated from these EVs and Next-generation sequencing was performed. The association between miR-126 levels and sepsis outcomes such as mortality, ARDS and ARF was analyzed.

**Results:**

Septic patients with circulating EVs that induced EC injury (lower transendothelial electrical resistance) were more likely to experience ARDS (p<0.05). Higher caspase-1 activity in total EVs, monocyte- or EC-derived EVs was significantly associated with the development of ARDS (p<0.05). MiR-126-3p levels in EC EVs were significantly decreased in ARDS patients compared with healthy controls (p<0.05). Moreover, a decline in miR-126-5p levels from day 1 to day 3 was associated with increased mortality, ARDS and ARF; while decline in miR-126-3p levels from day 1 to day 3 was associated with ARDS development.

**Conclusions:**

Enhanced caspase-1 activity and declining miR-126 levels in circulating EVs are associated with sepsis-related organ failure and mortality. Extracellular vesicular contents may serve as novel prognostic biomarkers and/or targets for future therapeutic approaches in sepsis.

## 1 Introduction

Sepsis remains a leading cause of death in intensive care units (ICU), with a mortality rate of approximately 15-25% (1–3). Vascular dysfunction, a feared complication of sepsis, can result in acute respiratory distress syndrome (ARDS), acute renal failure (ARF), and even mortality (4, 5). However, existing prognostic tools are hindered by complexity and biases leading to imperfect predictive capabilities (6–9). Further, the heterogeneity of sepsis at the individual patient level has also hindered therapeutic advances in the field (10). Decades of investigative efforts in the absence of sepsis endotyping have yielded no conclusively effective therapies to date. Understanding the heterogeneity of sepsis and subgrouping septic patients for personalized treatment may hold the promise of targeted sepsis therapy.

Extracellular vesicles (EVs) are membranous nanovesicles formed by outward budding of the plasma membrane, apoptosis, or exocytosis of multivesicular bodies, and are released from almost every cell type (11–13). EVs express molecules on their membranes that are specific for their parental cells and contain functional molecules including protein and miRNA (11). Circulating EVs encompass EVs from platelets, endothelial cells (ECs), neutrophils, monocytes, lymphocytes, erythrocytes, and their precursors, and play a critical role in maintaining vascular homeostasis in physiological conditions (13, 14). The presence of circulating EVs and their role as biomarkers and mediators in sepsis pathophysiology have been explored (11). Recent studies suggest that EV caspase-1 activity can induce EC injury and that sepsis-induced pyroptosis of monocytes and ECs can release caspase-1 containing EVs (15–18). Conversely, EV miR-126 has been shown to stabilize the endothelium in sepsis while modulating sepsis-related organ failure (19–22). However, the potential role of circulating total or cell-specific EVs in septic patients and their association with sepsis outcomes such as organ failure and mortality are still largely unknown.

We isolated circulating total and cell-specific EVs from septic patients and analyzed the association of EV function/contents with sepsis outcomes. Our data demonstrated that EV caspase-1 activity and miR-126 levels are associated with the development of organ failures and mortality and may serve as novel biomarkers and/or therapeutic targets for sepsis.

## 2 Materials and methods

### 2.1 Human donors

Septic (n=96) and non-septic critically ill (n=12) hospitalized patients were enrolled during 2015-2018 at the Medical University of South Carolina. We screened all admissions for the presence of sepsis based on the American College of Chest Physicians/Society of Critical Care Medicine consensus definition in use at the time of enrollment initiation (23). Additional inclusion criteria included age ≥18 years and admission into the hospital within the previous 24 hours. We excluded immunocompromised patients as defined by immunosuppressive medication use, leukopenia, current hematologic malignancy, and history of stem cell transplant. Additional exclusion criteria included: transfer in from other hospitals if subjects had spent > 24 hours in an ICU at the time of screening and care goals consistent with comfort measures only at the time of screening. Healthy control subjects (n=45) were recruited through local advertising. Informed consent for study participation and publication of results was obtained from all research subjects or their legally authorized representatives.

Organ failures, including the occurrence of ARDS or ARF, were adjudicated by study investigators using the Berlin definition and kidney disease: Improving Global Outcomes (KDIGO) criteria, respectively (24, 25). ICU and hospital length of stay, discharge destination and vital status were captured. Basic demographics were recorded from healthy controls. These studies were in accordance with the 1964 Helsinki declaration and its later amendments or comparable ethical standards and approved by the Institutional Review Board at the Medical University of South Carolina. The entire human cohort demographics are listed in **Table 1**.

**Table 1:** Demographic characteristics of cohort.

N=153	Healthy Control	Septic patients without ARDS	Septic patients with ARDS	non-septic critical illnesses
Subject size	45	75	21	12
Sex, Females (%),	34 (75.6%)	35 (46.7%)	8 (38.1%)	7 (58.3%)
Age ± SEM (years)	33.6 ± 2.0	58.5 ± 1.9	58.7 ± 3.8	52.3 ± 6.3
Race (%)				
White	31 (68.9%)	44 (58.7%)	14 (66.7%),	9 (75%)
Black	10 (22.2%)	26 (34.7%)	7 (33.3%)	3 (25%)
Other	2 (8.8%)	5 (6.6%)	0	0
Mortality rate (%)	N/A	9.3%	57.1%	25%

SEM, standard error of mean; N/A, not applicable.

### 2.2 Human plasma collection

Plasma samples were collected from septic subjects and non-septic critically ill subjects on day 1, and 3 after hospital admission, and from healthy controls. Peripheral blood was collected in tubes containing disodium EDTA, and then centrifuged at 2,000 x g for 15 minutes at 4°C to obtain the plasma. The collected plasma samples were aliquoted and stored at -80°C for further analysis.

### 2.3 Extracellular vesicle isolation and characterization

Circulating total EVs were isolated from frozen human plasma as described (26, 27). In brief, 1 ml of the collected plasma samples were centrifuged at 2,000 x g for 30 minutes at 4°C, the supernatant was further centrifuged at 12,000 x g for 30 minutes at 4°C. Then the supernatant was ultracentrifuged at 100,000 x g for 2 hours at 4°C to pellet the EVs. EVs pellet was washed once in PBS and ultracentrifuged again at 100,000 x g for 2 hours at 4°C. The pelleted EVs were then suspended in PBS and kept at -80°C for further analysis.

Circulating EVs derived from monocytes or endothelial cells were isolated as described by Goetzl et al. (28). Briefly, circulating total EVs were precipitated with ExoQuick (SBI). Monocyte cell-specific EVs were enriched by sequential immunoprecipitation with biotinylated anti-CD14 antibody (Thermo Fisher Scientific), and CD14^+^ EVs were considered monocyte-derived EVs. Endothelial cell-specific EVs were enriched by sequential immunoprecipitation with the biotinylated anti-CD31 (Thermo Fisher Scientific) and anti-CD146 antibodies (Novus Biologicals). CD31^+^/CD146^+^ EVs were considered endothelial cell-derived EVs.

EVs were isolated from 1 ml of human plasma, fixed with 4% paraformaldehyde and their morphology was detected using transmission electron microscopy in the Electron Microscopy Facility at the University of Connecticut Health Center. The size distribution and the total number of EVs were analyzed by nanoparticle tracking analysis (NTA) with ZetaView PMX 120 according to the manufacturer’s instructions (Particle Metrix, Meerbusch, Germany). The presence of specific EV markers (CD9, CD63 and CD81) and absence of common contaminants (Calnexin and GM130) were determined by western blot.

### 2.4 Cell culture

Human Lung Microvascular Endothelial Cells (HMVEC) were purchased from Lonza and cultured in EBM-2 supplemented with EGM-2^MV^ SingleQuot (Lonza, Allendale, NJ, USA) containing 5% FBS and 1% penicillin/streptomycin in a CO_2_ incubator with 5% CO_2_.

### 2.5 Transendothelial electrical resistance assay

1.5 x 10^3^ cells (HMVEC) were seeded into each well of 8 well arrays (8W10E+, Applied Biophysics) and cultured for 24 hours. Total circulating EVs at 1.5 x 10^10^/ml were added into each well and transendothelial electrical resistance (TEER) assays were performed using an ECIS instrument (Applied Biophysics). At 2 hours after EV addition, the resistance of HMVECs was compared to that of its starting point (%), recorded and analyzed.

### 2.6 Caspase-1 activity

Caspase-1 activity in circulating total EVs (1.5 x 10^10^ particles), monocyte-derived EVs (5.5 x 10^9^ particles) and EC-derived EVs (3 x 10^7^) was measured by a Caspase-Glo®1 Inflammasome Assay kit according to the instructions (Promega). The catalytically active caspase-1 was detected by caspase cleavage of selective caspase-1 substrate (Z-WEHD). In brief, we transferred the same amount of EVs in 100 µl PBS into the corresponding wells of a new white 96-well plate. We then added 100 µl of Caspase-Glo® 1 reagent and incubated the plate at room temperature for 1 hour before measuring luminescence using a plate-reader.

### 2.7 Real-Time RT-PCR

Total RNA was extracted from EC-specific EVs using the miRNeasy Serum/Plasma kit (Qiagen, Germantown, MD) according to the manufacturer’s instructions. For miRNA expression, the RNA was reverse transcribed using a QIAGEN miRNA Reverse Transcription Kit (Qiagen). An identical amount of *Caenorhabditis elegans* miR-39 was spiked into each sample to allow for the normalization of Cq values.

### 2.8 Western blot

EVs were lysed with ice-cold RIPA lysis buffer (Abcam) containing protease and phosphatase inhibitors (Cell Signaling). All lysed samples were kept on ice for 30 min and centrifuged for 10 min at 4 °C at 10,000×*g*. The cell lysates were collected, and protein concentrations were measured using a DC protein assay (Bio-Rad). A total of 20 μg of EV protein was loaded into each lane for western blot. All exosome-specific primary antibodies including the anti-CD9, anti-CD63 and the anti-CD81 antibodies (System Biosciences) were used at 1:1000 dilution, and exosome-validated peroxidase-labeled secondary antibody was used at 1: 10000 dilution. Anti-calnexin, anti-GM130, anti-beta-actin and anti-GAPDH antibodies were from Cell Signaling. The immunoreactive protein bands were visualized by ECL detection kit (GE Healthcare). The images of the Western blots are shown in **Supplementary Figures 1 and 2**.

### 2.9 Next-generation sequencing

EVs (approximately 10^12^ particles) were isolated from 500 μl of plasma taken on days 1 and 3 from subjects with sepsis or non-septic critical illnesses using qEV columns (Izon, Medford, MA). RNAs were isolated from plasma EVs using miRNeasy Serum/Plasma kit (Qiagen, Germantown, MD) according to the manufacturer’s instructions. Next-generation sequencing was performed at MUSC next-generation sequencing facility using standard protocols.

### 2.10 Data analysis

Statistical significance for caspase-1 activity or miR-126 levels in healthy controls, sepsis with ARDS, and non-ARDS septic patients were evaluated using analysis of variance (ANOVA). Associations between caspase-1 activity with the occurrence of mortality, ARDS and ARF among septic patients were evaluated using Wilcoxon rank sum tests. Associations between expression levels of EV miR-126-3p and miR-126-5p levels with mortality, ARDS, and ARF among septic patients were evaluated using a linear mixed model approach. Models included fixed effects for patient status (e.g. died vs. survived), day, and the interaction between patient status and day and a random subject effect to account for measures collected on the same patients. Differences between patient status over time were evaluated using linear contrasts from the model. P-values were Bonferroni adjusted for all comparisons considered. Analyses were conducted using GraphPad Prism and SAS software. A value of *p*<0.05 was considered statistically significant.

## 3 Results

### 3.1 Characterization of EVs

**Figure 1:**
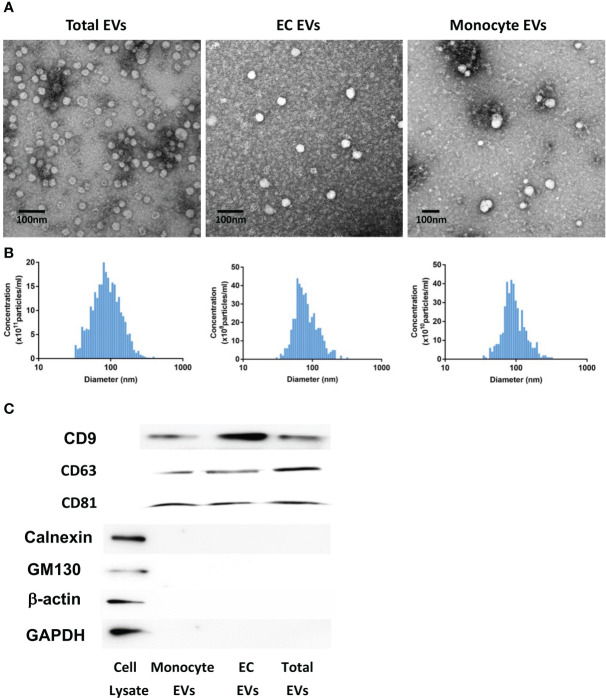
EV characterization. Analysis of EV morphology using transmission electron microscopy **(A)**. Scale bar: 100 nm. EV size distribution and concentration were measured by nanoparticle tracking analysis (NTA) with ZetaView **(B)**. The concentration of isolated total, monocyte-derived or EC-derived EVs was approximately 5.2 × 10^12^ particles/ml, 3.5 × 10^11^ particles/ml and 4.5 × 10^9^ particles/ml, respectively. All EVs showed a similar size distribution profile with isolated particles within the 30–120 nm range. EV markers (CD9, CD63 and CD81) and common contaminants (calnexin and GM130) in total, monocyte- and EC-derived EVs were detected by western blot **(C)**. EV, Extracellular vesicles. Cell Lysate: human brain pericytes.

We first characterized total, EC-derived or monocyte-derived EVs collected from plasma samples. Transmission electron microscopy was used to analyze EVs in all groups, and they exhibited typical morphology (**Figure 1A**). As shown in **Figure 1B**, the concentration of isolated total, EC-derived or monocyte-derived EVs was approximately 5.2 × 10^12^ particles/ml, 4.5 × 10^9^ particles/ml and 3.5 × 10^11^ particles/ml respectively. All EVs showed a similar size distribution profile with isolated particles within the 30–120 nm range (**Figure 1B**). Moreover, we observed that the total number of isolated EVs, including those derived from monocytes or endothelial cells, was similar between healthy controls and patients (data not shown). Western blot analysis validated the presence of EV markers CD9, CD63 and CD81 in total, monocyte- and EC-derived EVs, while common contaminants calnexin and GM130 were absent (**Figure 1C**). Additionally, both β-actin and GAPDH were not detected in total, monocyte-, or EC-derived EVs (**Figure 1C**).

### 3.2 The impact of circulating total EVs on vascular permeability is associated with the development of ARDS

**Figure 2:**
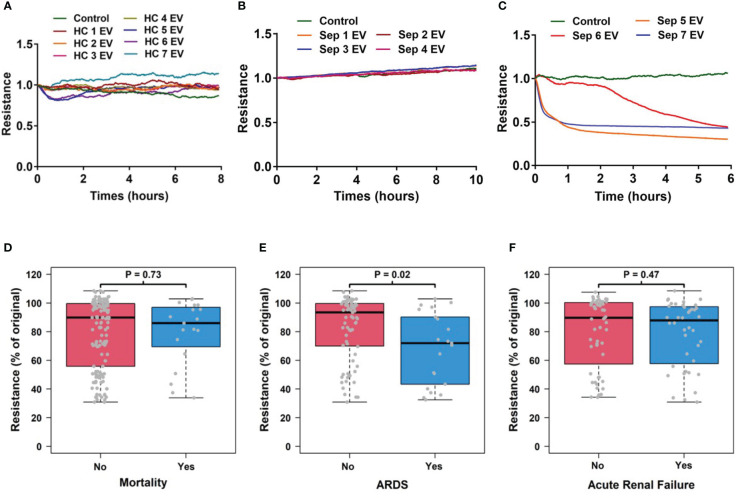
Circulating EV-induced vascular permeability is associated with ARDS patients. Circulating total EVs were isolated from healthy controls (N=7) and septic patients (N=96). Among septic patients, 21 patients developed ARDS and 75 did not. These EVs at 1.5 x 10^10^ EVs/ml were used in TEER assays which was measured in HMVEC for 6-24 hours by an ECIS instrument. Representative results from 7 healthy controls **(A)** and 7 septic patients **(B, C)** are shown. The association of TEER level with mortality **(D)**, ARDS development **(E)** and acute renal failure **(F)** were analyzed using Wilcoxon rank sum tests. Resistance levels are expressed as the level of impedance at 2 hours divided by the level of impedance prior to addition of EVs. Boxes present the 25^th^, 50^th^, and 75^th^ percentiles of resistance in each group. Whiskers extend 1.5 +/- the inner quartile range (i.e. difference between the 75^th^ and 25^th^ percentile). Grey points are the observed resistance values (% of original) for subjects within each group. EV, Extracellular vesicles; ARDS, acute respiratory distress syndrome; TEER, transendothelial electrical resistance; HMVEC, Human Lung Microvascular Endothelial Cell.

Since vascular injury plays a pivotal role in sepsis-related organ failures (4, 5), we first assessed the impact of circulating total EVs on vascular permeability *in vitro* using HMVECs and analyzed this impact’s association with sepsis outcomes including mortality, ARDS and ARF. We found that circulating EVs from healthy controls did not induce an increase in vascular permeability (n=7, **Figure 2A**). EVs from some septic patients did not induce vascular permeability (n=4, **Figure 2B**) while EVs of the same concentration from other septic patients induced increased vascular permeability (n=3, **Figure 2C**). Interestingly, total circulating EVs from sepsis survivors (n=73) versus non-survivors (n=19) showed no significantly different impact on transendothelial resistance (**Figure 2D**). In contrast, a significantly lower transendothelial resistance was induced by EVs from septic patients with ARDS (n=21) compared to those without ARDS (n=71) (p=0.02, **Figure 2E**). Circulating total EVs from septic patients with ARF (n=40) compared to those without ARF (n=52) showed no difference in their impact on transendothelial resistance (**Figure 2F**). These data demonstrate that ARDS is associated with the presence of circulating EVs which induce EC permeability.

### 3.3 Circulating total EV caspase-1 activity is associated with the development of ARDS

**Figure 3:**
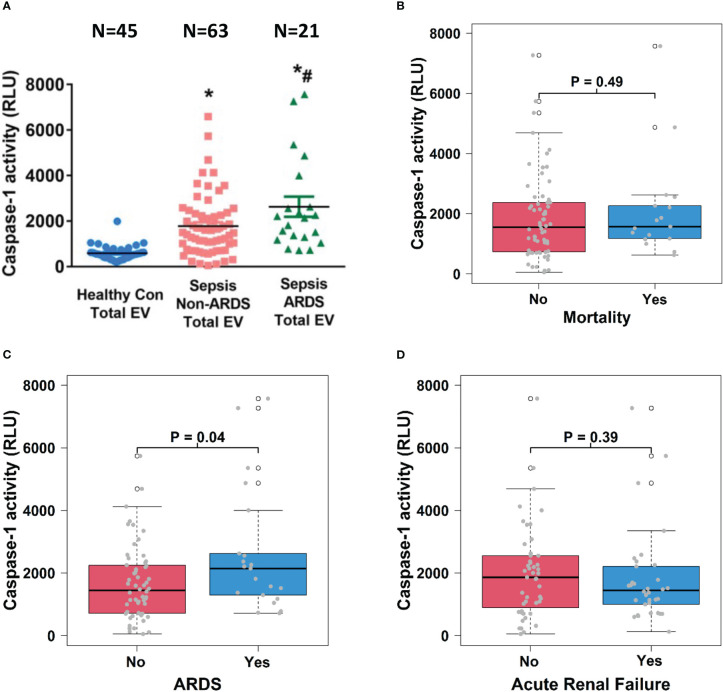
Circulating total EV caspase-1 activity is associated with ARDS development. Total plasma EVs were isolated from 45 healthy controls and 84 septic patients (21 ARDS and 63 non-ARDS), and caspase-1 activity **(A)** was determined. ^*^p < 0.05 compared to healthy controls, ^#^p<0.05 compared to non-ARDS septic patients. N=21-63/group. Statistical significance was determined by analysis of variance (ANOVA). The association of caspase-1 activity with mortality **(B)**, ARDS development **(C)** and acute renal failure **(D)** were analyzed using Wilcoxon rank sum tests. Boxes present the 25^th^, 50^th^, and 75^th^ percentiles of caspase-1 activity in each group. Whiskers extend 1.5 +/- the inner quartile range (i.e. difference between the 75^th^ and 25^th^ percentile). Grey points are the caspase-1 activity values for subjects within each group. EV, Extracellular vesicles; ARDS, acute respiratory distress syndrome.

To explore the relationship between EV content and sepsis outcomes, we determined caspase-1 activity in total circulating EVs from healthy controls and septic patients and analyzed its association with sepsis outcomes including mortality, ARDS and ARF. We found that caspase-1 activity was significantly increased in EVs from septic patients compared to those from healthy controls (p<0.05) and that among septic patients, EVs from patients who experienced ARDS (n=21) contained significantly increased caspase-1 activity than EVs from patients without ARDS (n=63) (p<0.05, **Figure 3A**). Circulating total EV caspase-1 activity from sepsis survivors (n=64) and non-survivors (n=17) showed no significant difference (**Figure 3B**). In contrast, a significantly higher total EV caspase-1 activity from septic patients with ARDS (n=21) compared to non-ARDS septic patients (n=60) was observed (p<0.05, **Figure 3C**). Circulating total EV caspase-1 activity from septic patients with ARF (n=34) and without ARF (n=47) was not significantly different (**Figure 3D**). These data demonstrate that circulating total EV caspase-1 activity is associated with ARDS development during sepsis, but not with mortality or ARF of sepsis.

### 3.4 Circulating monocyte-derived EV caspase-1 activity is associated with the development of ARDS

**Figure 4:**
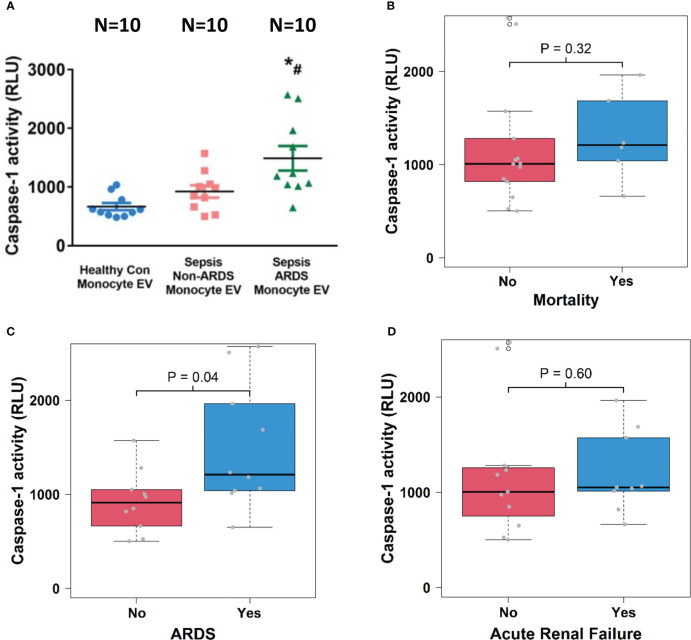
Circulating monocyte-derived EV caspase-1 activity is associated with ARDS development. Circulating monocyte-derived EVs were isolated from 10 healthy controls and 20 septic patients (10 ARDS and 10 non-ARDS), and caspase-1 activity **(A)** was determined. ^*^p < 0.05 compared to healthy controls, ^#^p<0.05 compared to non-ARDS septic patients. N=10/group. Statistical significance was determined by analysis of variance (ANOVA). The association of caspase-1 activity with mortality **(B)**, ARDS development **(C)** and acute renal failure **(D)** were analyzed using Wilcoxon rank sum tests for the analysis. Boxes present the 25^th^, 50^th^, and 75^th^ percentiles of caspase-1 activity in each group. Whiskers extend 1.5 +/- the inner quartile range (i.e. difference between the 75^th^ and 25^th^ percentile). Grey points are the caspase-1 activity values for subjects within each group. EV, Extracellular vesicles; ARDS, acute respiratory distress syndrome.

To explore the relationship between cell-specific EV content and sepsis outcomes, we examined caspase-1 activity in circulating monocyte-derived EVs. We found that caspase-1 activity was significantly increased in monocyte-derived EVs from septic patients with ARDS compared to those from healthy controls and septic patients without ARDS (N=10; p<0.05, **Figure 4A**). Monocyte-derived EV caspase-1 activity from sepsis survivors (n=14) and non-survivors (n=6) showed no significant difference (**Figure 4B**). In contrast, a significantly higher monocyte-derived EV caspase-1 activity from septic patients with ARDS (n=10) compared to non-ARDS septic patients (n=10) was observed (p<0.05, **Figure 4C**). Additionally, monocyte-derived EV caspase-1 activity from septic patients with (n=9) and without (n=11) ARF was comparable (**Figure 4D**). These data demonstrate that monocyte-derived EV caspase-1 activity is associated with ARDS development during sepsis, but not with mortality or ARF of sepsis

### 3.5 Circulating EC-derived EV caspase-1 activity is associated with the development of ARDS

**Figure 5:**
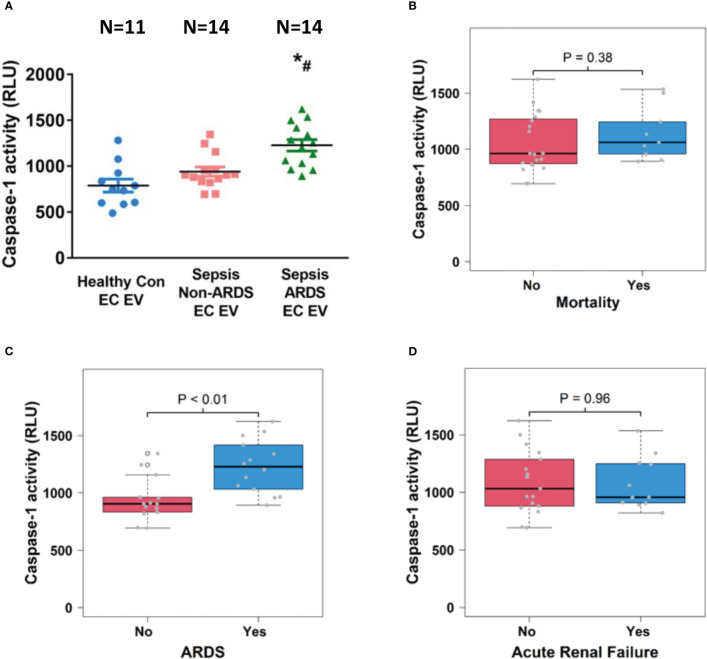
Circulating endothelial cell-derived EV caspase-1 activity is associated with ARDS development. Circulating endothelial cell-derived EVs were isolated from 11 healthy controls and 28 septic patients (14 ARDS and 14 non-ARDS), and caspase-1 activity **(A)** was determined. ^*^p < 0.05 compared to healthy controls, ^#^p<0.05 compared to non-ARDS septic patients. N=11-14/group. Statistical significance was determined by analysis of variance (ANOVA). The association of caspase-1 activity with mortality **(B)**, ARDS development **(C)** and acute renal failure **(D)** were analyzed using Wilcoxon rank sum tests. Boxes present the 25^th^, 50^th^, and 75^th^ percentiles of caspase-1 activity in each group. Whiskers extend 1.5 +/- the inner quartile range (i.e. difference between the 75^th^ and 25^th^ percentile). Grey points are the caspase-1 activity values for subjects within each group. EV, Extracellular vesicles; ARDS, acute respiratory distress syndrome.

We further examined caspase-1 activity in circulating EC-derived EVs and analyzed its association with sepsis outcomes including mortality, ARDS and ARF. We found that caspase-1 activity was significantly increased in EC-derived EVs from ARDS patients (n=14) compared to those from healthy controls (n=11) and non-ARDS septic patients (n=14; p<0.05, **Figure 5A**). Interestingly, EC-derived EV caspase-1 activity from sepsis survivors (n=19) and non-survivors (n=9) showed no significant difference (**Figure 5B**). In contrast, a significantly higher EC-derived EV caspase-1 activity from septic patients with ARDS (n=14) compared to non-ARDS septic patients (n=14) was observed (p<0.01, **Figure 5C**). Additionally, EC-derived EV caspase-1 activity from septic patients with ARF (n =11) and without (n=17) ARF was comparable (**Figure 5D**). These data demonstrate that EC-derived EV caspase-1 activity is associated with ARDS development during sepsis, but not with mortality or ARF of sepsis.

### 3.6 Circulating EC-derived EV miR-126-3p levels were decreased in ARDS patients

**Figure 6:**
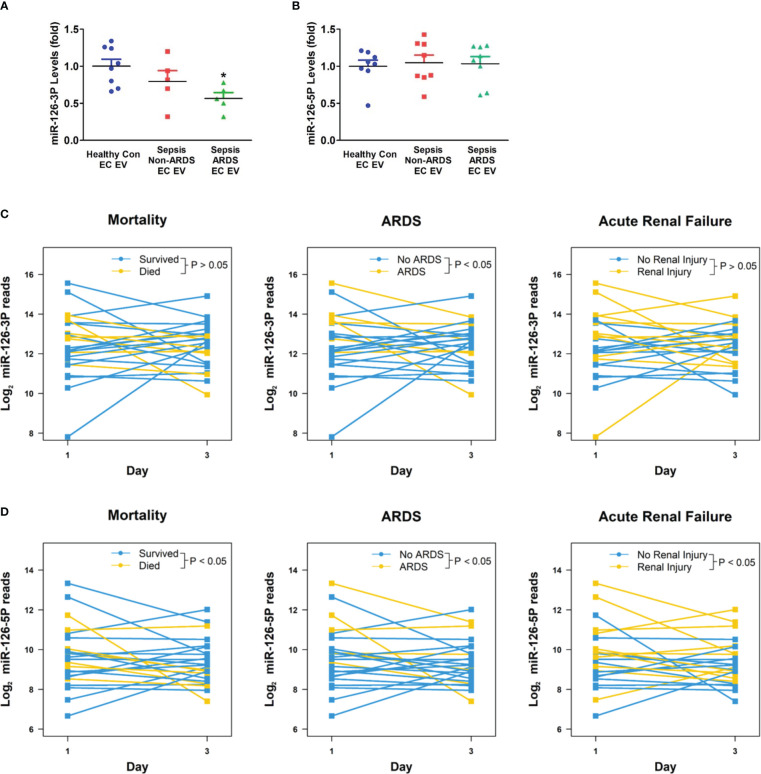
Circulating EV miR-126 levels and its association with clinical outcomes of sepsis. Circulating endothelial-cell-derived EVs were isolated, miRNA-126 levels **(A, B)** were determined in healthy controls, non-ARDS septic patients, and ARDS patients. *p < 0.05 compared to healthy controls. N=5-8/group. Statistical significance was determined by analysis of variance (ANOVA). Circulating total EV miRNA levels in sepsis and ICU control patients were determined by next generation sequencing. Association between EV miR-126-3p **(C)** or miR-126-5p **(D)** levels with sepsis outcomes (mortality; ARDS; acute renal failure) were analyzed using a linear mixed model approach. Differences between patient statuses over time were evaluated using linear contrasts from the model. P-values were Bonferroni adjusted for all comparisons considered. N=12/group. EV, Extracellular vesicles.

In addition to caspase-1, miR-126 has been implicated in vascular injury during sepsis and is one of the most abundant miRNAs in the EC-derived EVs (18, 20, 21). Thus, we next determined miR-126 levels in circulating EC-derived EVs from day 1 plasma samples. MiR-126-3p levels were significantly decreased in ARDS patients (n=5) compared to healthy control group (n=8; p<0.05, **Figure 6A**), while miR-126-5p levels were not different among the investigated groups (n=8, **Figure 6B**).

### 3.7 Circulating total EV miR-126 levels are associated with sepsis outcomes

To further analyze the relationship between EV miR-126 levels and sepsis outcomes, RNAs were isolated from circulating total EVs taken on days 1 and 3 from subjects with sepsis and non-septic critical illnesses controls. Next-generation sequencing identified that 51 miRNAs were differentially expressed between the two groups with miR-126-3p and -5p being among the most differentially expressed (adjusted p values of 0.03 and 0.003, respectively; data not shown). The temporary decline of EV miR-126-3p levels from day 1 to day 3 was significantly associated with ARDS development but not mortality or ARF (n=12, **Figure 6C**). An overall decline in EV miR-126-5p levels from day 1 to day 3 is significantly associated with increased mortality, ARDS and ARF (n=12, **Figure 6D**).

## 4 Discussion

Currently, there are no reliable biomarkers to predict sepsis outcomes. Caspase-1 and miR-126 in EVs have been implicated in lung injury and sepsis (18, 20, 21). In the present study, our findings demonstrated previously unappreciated associations of circulating EV caspase-1/miR-126 levels with sepsis outcomes. First, the impact of circulating total EVs on endothelial injury is associated with ARDS development during sepsis; second, caspase-1 activity in circulating EVs an associated with ARDS development during sepsis; third, a decline in total EV miR-126-5p levels from day 1 to day 3 is associated with mortality, ARDS and ARF during sepsis. Therefore, circulating EV function/contents may predict patient outcomes at an early time point, and lead to early clinical interventions to improve outcomes of septic patients.

During sepsis, leukocytes, endothelial cells, and platelets produce large amounts of EVs, which carry a variety of RNAs and proteins to modulate recipient cell function. Circulating EVs can be released and taken up by recipient cells quickly, thus, the pool of circulating EVs is dynamic and reflective of the temporal changes in cellular activation (11). Accordingly, the EV profile of each individual may change throughout the course of sepsis offering a window into the longitudinal states of cellular activation. A deeper understanding of circulating total/cell-specific EVs and their function/contents over the course of sepsis development may pave the way to identify novel biomarkers and subgroup septic patients for future personalized treatment.

EC dysfunction, manifested by increases in vascular permeability, is an important feature of sepsis, and plays a critical role in the pathogenesis of multi-organ failure (5, 29, 30). Platelet-derived EVs from septic patients have been reported to induce endothelial cell apoptosis (31). However, the role of circulating EVs from patients with sepsis on endothelial dysfunction, and their connections with sepsis outcomes remain largely unknown. Our data demonstrated that circulating EVs from a subset of septic patients induce vascular permeability while EVs from others did not. Interestingly, septic patients with circulating EVs that induced vascular permeability were more likely to develop ARDS compared to those patients who did not, suggesting that the impact of circulating EVs on vascular injury may contribute to ARDS development during sepsis. Thus, a deeper understanding of circulating EV properties and biology may allow for sepsis endotyping and lead to novel therapeutic strategies.

Caspase-1, a member of the cysteinyl proteases family, plays a critical role in regulating inflammatory responses, apoptosis, and pyroptosis, three known features of sepsis (32–35). A growing body of evidence has confirmed that inhibiting caspase-1 is beneficial in sepsis, as it can reduce inflammation, apoptosis and pyroptosis, and attenuate organ injury (34, 36–39). In addition, circulating EVs isolated from septic patients induce lymphocyte apoptosis via caspase-1 (33). However, circulating EV caspase-1 and its association with sepsis outcomes remains largely unknown. In agreement with previous findings that circulating EV caspase-1 activity was significantly enhanced in septic patients compared with controls (33), we further demonstrated that caspase-1 activity in circulating EVs was significantly higher in septic patients who experienced ARDS as compared to those who did not. This observation was further seen in EVs derived from both monocytes and endothelial cells suggesting a potentially important role of these cells in cellular cross-talk in the setting of sepsis-related organ failure. This is consistent with previous findings that EVs from cultured monocytes can cause lung vascular endothelial cell injury via its caspase-1 content (17, 18). Thus, circulating monocyte EV caspase-1 may induce vascular injury and contribute to ARDS development during sepsis. These initial studies demonstrate the feasibility of using cell-specific EV caspase-1 to develop novel biomarkers and subgroup septic patients.

Our previous studies suggested that EC EV miR-126 improves the outcomes of lipopolysaccharide-induced acute lung injury and cecal ligation and puncture-induced sepsis via reducing inflammation and vascular permeability in an animal model (20, 21). Recent studies have further confirmed that delivering miR-126 to septic animals can alleviate multiple organ dysfunction including lung, liver, kidney, brain, and heart by reducing inflammation and vascular injury (19, 22, 40). However, circulating EV miR-126 content and its association with human sepsis outcomes remains largely unknown. Here we extended previous observations that serum miR-126-3p levels were decreased among septic patients compared to control subjects (41) by demonstrating that circulating EV miR-126-3p levels are significantly decreased in septic patients who experience ARDS as compared to healthy controls. In addition, we demonstrated for the first time that a decline in circulating total EV miR-126-5p levels is associated with mortality, ARDS and ARF while decreased miR-126-3p levels is associated with ARDS development during sepsis. These data indicate that further investigations on circulating EV miR-126 levels may provide novel biomarkers or therapeutic targets for sepsis.

There are several limitations of this study. First, we detected the effect of circulating total EVs on EC permeability and its association with sepsis outcomes; however, the effect of cell-specific EVs from platelets, ECs, neutrophils, monocytes, and other cell types on EC permeability remains to be further investigated. Second, we examined caspase-1 levels in monocyte- or EC-derived EVs and their association with clinical features in a small cohort of septic patients. However, further validation studies in larger cohorts of septic patients are necessary. Last, the association of circulating total EV miR-126 levels with sepsis outcomes has been determined in this study; however, cell-specific EV miR-126 levels in a large cohort of septic patients and their connections to sepsis outcomes need further investigation.

In summary, we discovered novel associations between circulating EV function/contents and sepsis outcomes, which may lead to the discovery of novel biomarkers for sepsis and possible new therapeutic approaches.

## Data availability statement

The raw data supporting the conclusions of this article will be made available by the authors, without undue reservation.

## Ethics statement

The studies involving human participants were reviewed and approved by the Institutional Review Board at the Medical University of South Carolina. The patients/participants provided their written informed consent to participate in this study.

## Author contributions

PL performed experiments, data collection and analysis, and wrote and edited the manuscript. YW performed experiments, collected data, and edited the manuscript. AG enrolled and collected data from sepsis subjects, performed experiments, and edited the manuscript. BW helped with data analysis and manuscript editing. PH helped with study design and manuscript editing. HW helped with study design and manuscript editing. BZ helped with study design and manuscript editing. HF was responsible for study conception and design, data analysis and interpretation, study supervision, and manuscript writing and editing. All authors have read and approved of this manuscript. All authors contributed to the article.

## References

[1] Rudd KE Johnson SC Agesa KM Shackelford KA Tsoi D Kievlan DR. Global, regional, and national sepsis incidence and mortality, 1990-2017: analysis for the global burden of disease study. Lancet (2020) 395(10219):200–11. doi: 10.1016/S0140-6736(19)32989-7 PMC6970225 31954465

[2] Fidalgo P Nora D Coelho L Povoa P. Pancreatic stone protein: review of a new biomarker in sepsis. J Clin Med (2022) 11(4):1085. doi: 10.3390/jcm11041085 PMC8880320 35207355

[3] Singer M Deutschman CS Seymour CW Shankar-Hari M Annane D Bauer M. The third international consensus definitions for sepsis and septic shock (Sepsis-3). JAMA (2016) 315(8):801–10. doi: 10.1001/jama.2016.0287 PMC4968574 26903338

[4] De Backer D Orbegozo Cortes D Donadello K Vincent JL. Pathophysiology of microcirculatory dysfunction and the pathogenesis of septic shock. Virulence (2014) 5(1):73–9. doi: 10.4161/viru.26482 PMC3916386 24067428

[5] Darwish I Liles WC. Emerging therapeutic strategies to prevent infection-related microvascular endothelial activation and dysfunction. Virulence (2013) 4(6):572–82. doi: 10.4161/viru.25740 PMC5359747 23863603

[6] Sarkar R Martin C Mattie H Gichoya JW Stone DJ Celi LA. Performance of intensive care unit severity scoring systems across different ethnicities in the USA: a retrospective observational study. Lancet Digit Health (2021) 3(4):e241–e9. doi: 10.1016/S2589-7500(21)00022-4 PMC8063502 33766288

[7] Miller WD Han X Peek ME Charan Ashana D Parker WF. Accuracy of the sequential organ failure assessment score for in-hospital mortality by race and relevance to crisis standards of care. JAMA Netw Open (2021) 4(6):e2113891. doi: 10.1001/jamanetworkopen.2021.13891 34143190 PMC8214156

[8] Brinton DL Ford DW Martin RH Simpson KN Goodwin AJ Simpson AN. Missing data methods for intensive care unit sofa scores in electronic health records studies: results from a Monte Carlo simulation. J Comp Eff Res (2022) 11(1):47–56. doi: 10.2217/cer-2021-0079 34726477

[9] Ashana DC Anesi GL Liu VX Escobar GJ Chesley C Eneanya ND. Equitably allocating resources during crises: racial differences in mortality prediction models. Am J Respir Crit Care Med (2021) 204(2):178–86. doi: 10.1164/rccm.202012-4383OC PMC8759151 33751910

[10] Stanski NL Wong HR. Prognostic and predictive enrichment in sepsis. Nat Rev Nephrol (2020) 16(1):20–31. doi: 10.1038/s41581-019-0199-3 31511662 PMC7097452

[11] Raeven P Zipperle J Drechsler S. Extracellular vesicles as markers and mediators in sepsis. Theranostics (2018) 8(12):3348–65. doi: 10.7150/thno.23453 PMC6010985 29930734

[12] van der Pol E Boing AN Gool EL Nieuwland R. Recent developments in the nomenclature, presence, isolation, detection and clinical impact of extracellular vesicles. J Thromb Haemost (2016) 14(1):48–56. doi: 10.1111/jth.13190 26564379

[13] Karasu E Eisenhardt SU Harant J Huber-Lang M. Extracellular vesicles: packages sent with complement. Front Immunol (2018) 9:721. doi: 10.3389/fimmu.2018.00721 29696020 PMC5904200

[14] Reid VL Webster NR. Role of microparticles in sepsis. Br J Anaesth (2012) 109(4):503–13. doi: 10.1093/bja/aes321 22952169

[15] Cheng KT Xiong S Ye Z Hong Z Di A Tsang KM. Caspase-11-Mediated endothelial pyroptosis underlies endotoxemia-induced lung injury. J Clin Invest (2017) 127(11):4124–35. doi: 10.1172/JCI94495 PMC5663346 28990935

[16] Wu C Lu W Zhang Y Zhang G Shi X Hisada Y. Inflammasome activation triggers blood clotting and host death through pyroptosis. Immunity (2019) 50(6):1401–11 e4. doi: 10.1016/j.immuni.2019.04.003 31076358 PMC6791531

[17] Mitra S Wewers MD Sarkar A. Mononuclear phagocyte-derived microparticulate caspase-1 induces pulmonary vascular endothelial cell injury. PloS One (2015) 10(12):e0145607. doi: 10.1371/journal.pone.0145607 26710067 PMC4692444

[18] Mitra S Exline M Habyarimana F Gavrilin MA Baker PJ Masters SL. Microparticulate caspase 1 regulates gasdermin d and pulmonary vascular endothelial cell injury. Am J Respir Cell Mol Biol (2018) 59(1):56–64. doi: 10.1165/rcmb.2017-0393OC 29365280 PMC6039876

[19] Zou Q Liu C Hu N Wang W Wang H. Mir-126 ameliorates multiple organ dysfunction in septic rats by regulating the differentiation of Th17/Treg. Mol Biol Rep (2022) 49(4):2985–98. doi: 10.1007/s11033-022-07121-w PMC8817156 35122598

[20] Zhou Y Li P Goodwin AJ Cook JA Halushka PV Chang E. Exosomes from endothelial progenitor cells improve outcomes of the lipopolysaccharide-induced acute lung injury. Crit Care (2019) 23(1):44. doi: 10.1186/s13054-019-2339-3 30760290 PMC6373158

[21] Zhou Y Li P Goodwin AJ Cook JA Halushka PV Chang E. Exosomes from endothelial progenitor cells improve the outcome of a murine model of sepsis. Mol Ther (2018) 26(5):1375–84. doi: 10.1016/j.ymthe.2018.02.020 PMC5993985 29599080

[22] Nong A Li Q Huang Z Xu Y He K Jia Y. Microrna mir-126 attenuates brain injury in septic rats Via nf-kappab signaling pathway. Bioengineered (2021) 12(1):2639–48. doi: 10.1080/21655979.2021.1937905 PMC8806573 34115555

[23] Bone RC Balk RA Cerra FB Dellinger RP Fein AM Knaus WA. Definitions for sepsis and organ failure and guidelines for the use of innovative therapies in sepsis. the Accp/Sccm consensus conference committee. American college of chest Physicians/Society of critical care medicine. Chest (1992) 101(6):1644–55. doi: 10.1378/chest.101.6.1644 1303622

[24] Force ADT Ranieri VM Rubenfeld GD Thompson BT Ferguson ND Caldwell E. Acute respiratory distress syndrome: the Berlin definition. JAMA (2012) 307(23):2526–33. doi: 10.1001/jama.2012.5669 22797452

[25] Khwaja A. Kdigo clinical practice guidelines for acute kidney injury. Nephron Clin Pract (2012) 120(4):c179–84. doi: 10.1159/000339789 22890468

[26] Osti D Del Bene M Rappa G Santos M Matafora V Richichi C. Clinical significance of extracellular vesicles in plasma from glioblastoma patients. Clin Cancer Res (2019) 25(1):266–76. doi: 10.1158/1078-0432.CCR-18-1941 30287549

[27] Thery C Amigorena S Raposo G Clayton A. Isolation and characterization of exosomes from cell culture supernatants and biological fluids. Curr Protoc Cell Biol (2006) 3.22.1-29. doi: 10.1002/0471143030.cb0322s30 18228490

[28] Abner EL Elahi FM Jicha GA Mustapic M Al-Janabi O Kramer JH. Endothelial-derived plasma exosome proteins in alzheimer's disease angiopathy. FASEB J (2020) 34(4):5967–74. doi: 10.1096/fj.202000034R PMC7233139 32157747

[29] Natarajan V Dudek SM Jacobson JR Moreno-Vinasco L Huang LS Abassi T. Sphingosine-1-Phosphate, Fty720, and sphingosine-1-Phosphate receptors in the pathobiology of acute lung injury. Am J Respir Cell Mol Biol (2013) 49(1):6–17. doi: 10.1165/rcmb.2012-0411TR 23449739 PMC3727889

[30] Trzeciak S Cinel I Phillip Dellinger R Shapiro NI Arnold RC Parrillo JE. Resuscitating the microcirculation in sepsis: the central role of nitric oxide, emerging concepts for novel therapies, and challenges for clinical trials. Acad Emerg Med (2008) 15(5):399–413. doi: 10.1111/j.1553-2712.2008.00109.x 18439194 PMC2727641

[31] Gambim MH do Carmo Ade O Marti L Verissimo-Filho S Lopes LR Janiszewski M. Platelet-derived exosomes induce endothelial cell apoptosis through peroxynitrite generation: experimental evidence for a novel mechanism of septic vascular dysfunction. Crit Care (2007) 11(5):R107. doi: 10.1186/cc6133 17894858 PMC2556756

[32] Gao YL Zhai JH Chai YF. Recent advances in the molecular mechanisms underlying pyroptosis in sepsis. Mediators Inflammation (2018) 2018:5823823. doi: 10.1155/2018/5823823 PMC5863298 29706799

[33] Exline MC Justiniano S Hollyfield JL Berhe F Besecker BY Das S. Microvesicular caspase-1 mediates lymphocyte apoptosis in sepsis. PloS One (2014) 9(3):e90968. doi: 10.1371/journal.pone.0090968 24643116 PMC3958341

[34] Matute-Bello G. Targeting caspase-1 in sepsis: a novel approach to an old problem. Intensive Care Med (2007) 33(5):755–7. doi: 10.1007/s00134-007-0589-z 17384934

[35] Nhan TQ Liles WC Schwartz SM. Physiological functions of caspases beyond cell death. Am J Pathol (2006) 169(3):729–37. doi: 10.2353/ajpath.2006.060105 PMC1698830 16936249

[36] Fu Q Wu J Zhou XY Ji MH Mao QH Li Q. Nlrp3/Caspase-1 pathway-induced pyroptosis mediated cognitive deficits in a mouse model of sepsis-associated encephalopathy. Inflammation (2019) 42(1):306–18. doi: 10.1007/s10753-018-0894-4 PMC6394578 30276509

[37] Xu XE Liu L Wang YC Wang CT Zheng Q Liu QX. Caspase-1 inhibitor exerts brain-protective effects against sepsis-associated encephalopathy and cognitive impairments in a mouse model of sepsis. Brain Behav Immun (2019) 80:859–70. doi: 10.1016/j.bbi.2019.05.038 31145977

[38] Yang M Fang JT Zhang NS Qin LJ Zhuang YY Wang WW. Caspase-1-Inhibitor Ac-Yvad-Cmk inhibits pyroptosis and ameliorates acute kidney injury in a model of sepsis. BioMed Res Int (2021) 2021:6636621. doi: 10.1155/2021/6636621 34222479 PMC8213477

[39] Sarkar A Hall MW Exline M Hart J Knatz N Gatson NT. Caspase-1 regulates escherichia coli sepsis and splenic b cell apoptosis independently of interleukin-1beta and interleukin-18. Am J Respir Crit Care Med (2006) 174(9):1003–10. doi: 10.1164/rccm.200604-546OC PMC2648100 16908867

[40] Zhang X Wang X Fan M Tu F Yang K Ha T. Endothelial Hspa12b exerts protection against sepsis-induced severe cardiomyopathy Via suppression of adhesion molecule expression by mir-126. Front Immunol (2020) 11:566. doi: 10.3389/fimmu.2020.00566 32411123 PMC7201039

[41] Chen C Zhang L Huang H Liu S Liang Y Xu L. Serum mir-126-3p level is down-regulated in sepsis patients. Int J Clin Exp Pathol (2018) 11(5):2605–12. PMC6958305 31938374

