# Supplementary material for: Circulating extracellular vesicles are associated with the clinical outcomes of sepsis
Source: Front Immunol. 2023 Apr 25;14:1150564. doi: 10.3389/fimmu.2023.1150564 (PMC10167034; doi:10.3389/fimmu.2023.1150564)
Supplement: Supplementary file 1 [file DataSheet_1.pdf]

Supplementary Material

Circulating extracellular vesicles are associated with the clinical outcomes of sepsis

Pengfei Li, Yan Wu, Andrew J. Goodwin, Bethany Wolf, Perry V. Halushka, Hongjun Wang, Basilia Zingarelli, and Hongkuan Fan*

*** Correspondence:** Hongkuan Fan: fanhong@musc.edu

1 Supplementary Figures

Supplementary Figure 1

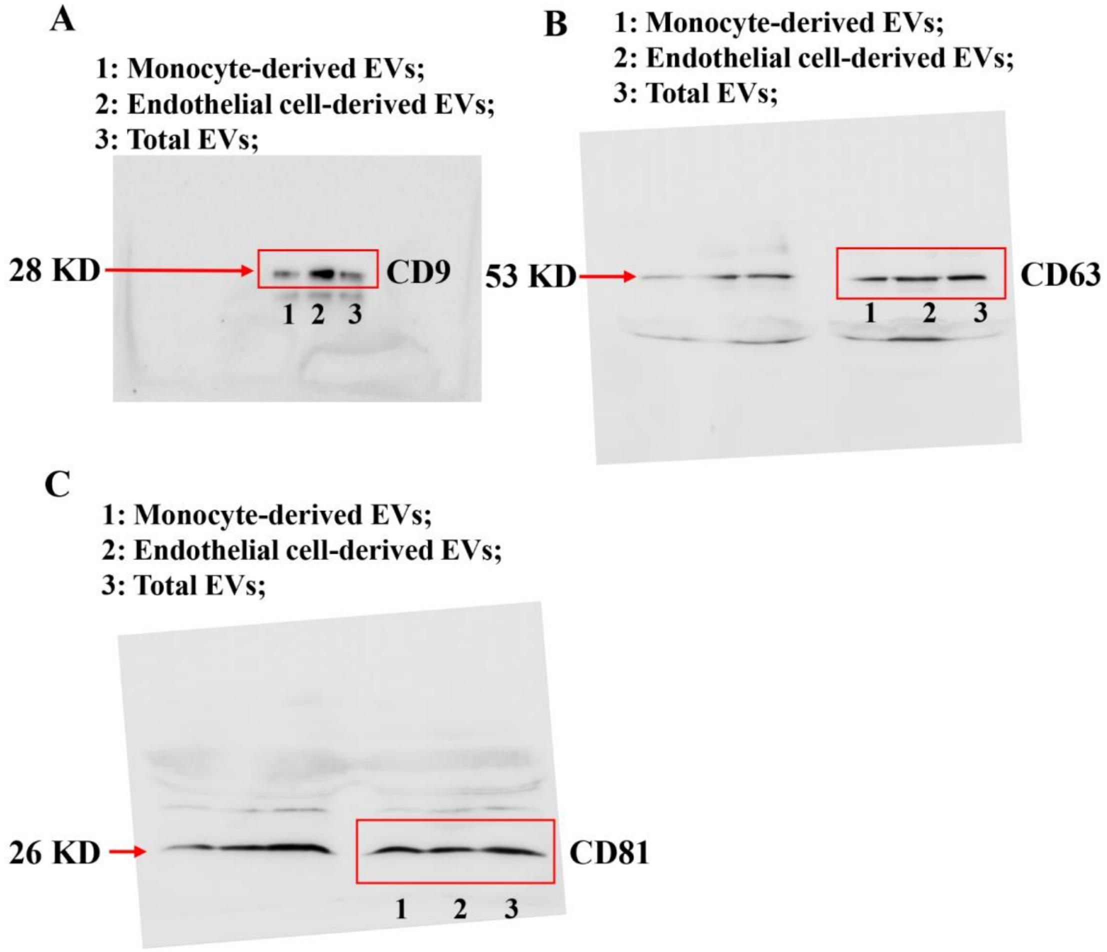

Supplementary Figure 1. The image of the Western blot for CD9 (A), CD63 (B) and CD81 (C) with molecular weight marker. 1: Monocyte-derived EVs, 2: Endothelial cell-derived EVs, 3: Total EVs.

Supplementary Figure 2

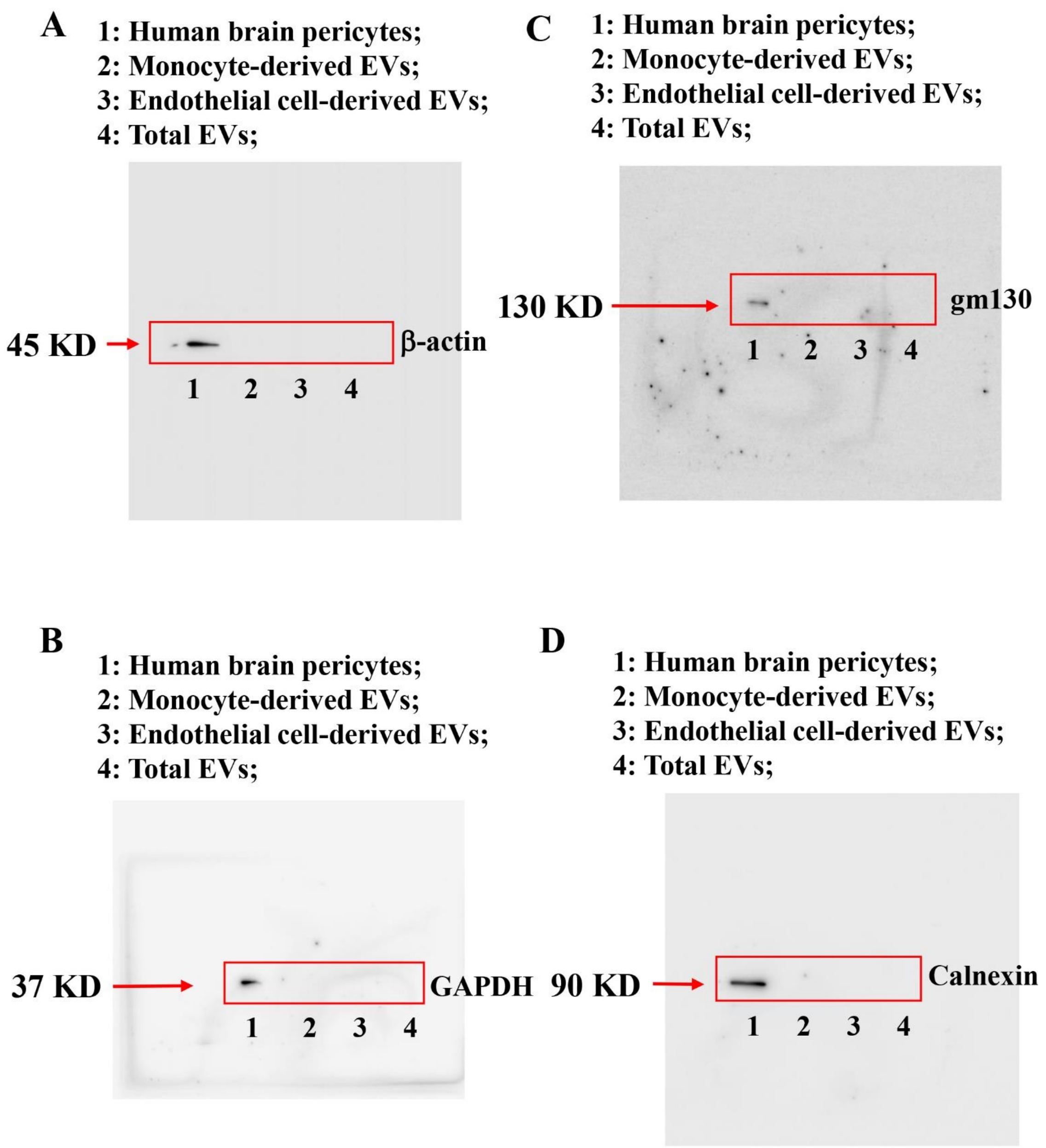

Supplementary Figure 2. The image of the Western blot for β-actin (A), GAPDH (B), gm130 (C), and Calnexin (D) with molecular weight marker. 1: Cell lysate from human brain pericytes, 2: Monocyte-derived EVs, 3: Endothelial cell-derived EVs, 4: Total EVs.
